# Alpha-synuclein-induced mitochondrial dysfunction is mediated via a sirtuin 3-dependent pathway

**DOI:** 10.1186/s13024-019-0349-x

**Published:** 2020-01-13

**Authors:** Jae-Hyeon Park, Jeremy D. Burgess, Ayman H. Faroqi, Natasha N. DeMeo, Fabienne C. Fiesel, Wolfdieter Springer, Marion Delenclos, Pamela J. McLean

**Affiliations:** 1 Department of Neuroscience, Mayo Clinic, 4500 San Pablo Road, Jacksonville, FL 32224 USA; 2 Neuroscience PhD Program, Mayo Clinic Graduate School of Biomedical Sciences, Mayo Clinic College of Medicine, 4500 San Pablo Road, Jacksonville, FL 32224 USA

**Keywords:** α-Synuclein, Sirtuin 3, Mitochondria dysfunction, Parkinson’s disease

## Abstract

**Background:**

Misfolding and aggregation of the presynaptic protein alpha-synuclein (αsyn) is a hallmark of Parkinson’s disease (PD) and related synucleinopathies. Although predominantly localized in the cytosol, a body of evidence has shown that αsyn localizes to mitochondria and contributes to the disruption of key mitochondrial processes. Mitochondrial dysfunction is central to the progression of PD and mutations in mitochondrial-associated proteins are found in familial cases of PD. The sirtuins are highly conserved nicotinamide adenine dinucleotide (NAD^+^)-dependent enzymes that play a broad role in cellular metabolism and aging. Interestingly, mitochondrial sirtuin 3 (SIRT3) plays a major role in maintaining mitochondrial function and preventing oxidative stress, and is downregulated in aging and age-associated diseases such as neurodegenerative disorders. Herein, we hypothesize that αsyn is associated with decreased SIRT3 levels contributing to impaired mitochondrial dynamics and biogenesis in PD.

**Methods:**

The level of mitochondrial SIRT3 was assessed in cells expressing oligomeric αsyn within the cytosolic and mitochondrial-enriched fractions. Mitochondrial integrity, respiration, and health were examined using several markers of mitochondrial dynamics and stress response and by measuring the rate of oxygen consumption (OCR). Our findings were validated in a rodent model of PD as well as in human post-mortem Lewy body disease (LBD) brain tissue.

**Results:**

Here, we demonstrate that αsyn associates with mitochondria and induces a decrease in mitochondrial SIRT3 levels and mitochondrial biogenesis. We show that SIRT3 downregulation is accompanied by decreased phosphorylation of AMPK and cAMP-response element binding protein (CREB), as well as increased phosphorylation of dynamin-related protein 1 (DRP1), indicative of impaired mitochondrial dynamics. OCR was significantly decreased suggesting a mitochondria respiratory deficit. Interestingly treatment with AMPK agonist 5-aminoimidazole-4-carboxamide-1-β-d-ribofuranoside (AICAR) restores SIRT3 expression, improves mitochondrial function, and decreases αsyn oligomer formation in a SIRT3-dependent manner.

**Conclusions:**

Together, our findings suggest that pharmacologically increasing SIRT3 levels can counteract αsyn-induced mitochondrial dysfunction by reducing αsyn oligomers and normalizing mitochondrial bioenergetics. These data support a protective role for SIRT3 in PD-associated pathways and contribute significant mechanistic insight into the interplay of SIRT3 and αsyn.

## Background

Alpha-synuclein (αsyn) accumulation is believed to be a key step in the pathogenesis of Parkinson’s disease (PD) and related alpha-synucleinopathies. Despite predominant localization in the cytosol, αsyn is found localized to mitochondria in post-mortem PD brain [1]. Mitochondrial accumulation of αsyn has been associated with impaired complex-I dependent respiration, decreased mitochondrial membrane potential, and increased levels of mitochondrial reactive oxygen species (mtROS) in multiple cellular models [1–4]. The evidence supporting the contribution of abnormal accumulation of αsyn to disruption of mitochondrial processes is compelling and indicates a crucial role for αsyn-induced mitochondrial dysfunction in PD pathogenesis and alpha-synucleopathies.

The sirtuins (SIRTs) are a family of nicotinamide adenine dinucleotide (NAD^+^)-dependent deacetylases and/or adenosine diphosphate (ADP)-ribosyltransferases that have long been recognized as essential for cell survival, metabolism, and longevity [5]. In mammals there are seven human SIRT homologs (SIRT1–7) with varied enzymatic activities. SIRT1, SIRT6, and SIRT7 predominantly reside in the nucleus whereas SIRT2 is located in the cytoplasm, and SIRT3, 4, and 5 reside in the mitochondria. SIRTs have been previously implicated in mechanisms of PD in a number of in vitro and in vivo studies [6–8]. The use of pharmacological activators and inhibitors of SIRTs in PD models have revealed neuroprotective and beneficial effects. For example, resveratrol, an activator of SIRT1, protects against cell death in neurotoxin-induced PD animal models [9, 10] and we have shown that SIRT2 inhibitors can rescue αsyn-mediated toxicity in cellular PD models [8]. SIRT3 is the predominant mitochondrial sirtuin and the major regulator of mitochondrial protein acetylation [11–13]. SIRT3 is expressed at high levels in the brain [14, 15] and plays an important role in maintaining mitochondrial integrity, energy metabolism, and regulating mitochondrial oxidative pathways [16, 17]. SIRT3-mediated deacetylation activates enzymes responsible for the reduction of ROS production, such as superoxide dismutase 2 (SOD2) [18]. Interestingly, SIRT3 acts as a pro-survival factor in neurons exposed to excitotoxic injury [19] and recent studies demonstrate a neuroprotective effect of SIRT3 in cell culture models of stroke, Huntington’s disease (HD), and Alzheimer’s disease (AD) [20–22]. Importantly, and relevant to the present study, overexpression of SIRT3 was recently demonstrated to prevent dopaminergic cell loss in a rodent model of PD [11].

Experimental evidence supports SIRT3-induced protection against oxidative stress by enhancement of mitochondrial biogenesis and integrity [23]. The multifaceted mitochondrial health-enhancing capabilities of SIRT3 thus make it an attractive therapeutic target for neurodegenerative diseases where mitochondrial dysfunction contributes to disease pathogenesis. Herein, we investigate a role for SIRT3 in PD pathogenesis and identify a potential mechanistic interaction between SIRT3 and αsyn. We hypothesize that the association of αsyn with mitochondria reduces SIRT3 deacetylase activity and contributes to mitochondrial dysfunction and pathogenesis in PD and related alpha-synucleinopathies. The data presented herein significantly advances our mechanistic understanding of SIRT3 in mitochondrial dysfunction and validates a protective role for SIRT3 in PD. Overall we confirm the potential application of SIRT3 activators as future targets for pharmacological strategies against neurodegeneration in PD and related alpha-synucleinopathies.

## Methods

### Cell culture

A stable cell line co-expressing human αsyn fused to either the amino-terminal (SL1) or carboxy-terminal fragment (SL2) of humanized *Gaussia princeps* luciferase was generated and described previously [24]. H4 SL1&SL2 and wt-αsyn cells were maintained at 37 °C in a 95% air/5% CO_2_ humidified incubator in Opti-MEM supplemented with 10% FBS. Stock cultures were kept in the presence of 1 μg/ml tetracycline (Invitrogen) to block the expression of the transgenes (SL1&SL2, wt--αsyn). αSyn expression is turned on or off by the absence (Tet- cells) or presence (Tet + cells) of tetracycline respectively.

Embryonic primary cortical neurons were prepared from E15 CD1 wildtype mice (Charles River, Wilmington, MA). Briefly, brains of E15 embryos were dissected in calcium and magnesium free HBSS, dissociated with 0.25% trypsin-EDTA (Life Technologies, Grand Island, NY), and seeded on poly-d-lysine coated 6 cm dishes at 0.95 × 10^5^ cells per cm^2^ (2 × 10^6^ cells per dish) in Neurobasal media containing 10% FBS, 1% pen/strep and 1% glutamax. After 1 h, media was exchanged for Neurobasal containing B-27 supplement, 1% pen/strep and 1% glutamax. Neurons were maintained at 37 °C in a humidified incubator with 5% CO_2_/95% air. At day 7 in vitro (DIV) neurons were transduced with adeno-associated-virus (AAV) serotype2/8 expressing wt-αsyn or venusYFP under the chicken beta actin promoter.

### Rodent stereotaxic surgery

Adult female Sprague Dawley rats (225-250 g, Envigo, USA) were housed and treated in accordance with the NIH Guide for Care and Use of Laboratory animals. All animal procedures were approved by the Mayo Institutional Animal Care and Use Committee and are in accordance with the NIH Guide for Care and Use of Laboratory animals. All viral vector delivery surgical procedures and tissue processing was performed as previously described by our group [25]. Briefly, AAVs serotype 2/8 expressing human αsyn fused with either the C-terminus (AAV-SL1) or N-terminus (AAV-SL2) of *Gaussia princeps* luciferase were produced by plasmid triple transfection with helper plasmids in HEK293T cells. 48 h later, cells were harvested and lysed in the presence of 0.5% sodium deoxycholate and 50 U/ml Benzonase (Sigma-Aldrich, St. Louis, MO) by freeze-thawing, and the virus was isolated using a discontinuous iodixanol gradient. The genomic titer of each virus was determined by quantitative PCR. A combination of AAV-SL1 (8.10e12gc/ml) + AAV-SL2 (8.10e12 gc/ml) was delivered directly to the right substantia nigra/midbrain (SN) using stereotaxic surgery (coordinates: AP − 5.2 mm, ML + 2.0 mm, DV + 7.2 mm from dura) [26]. A mix of AAVs were infused at a rate of 0.4 μL/min (final volume 2 μL) using a microinjector (Stoelting). A group of control animals were injected with 2 μL of AAV8 expressing full length of humanized *Gaussia princeps* luciferase (AAV8-Hgluc).

### Human brain tissue

Frozen human post-mortem brain was provided by the Mayo Clinic brain bank at the Mayo Clinic in Jacksonville. For this study, striatum (STR) samples from 10 control patients (6 females, 4 males) and 10 patients diagnosed with Lewy body disease (LBD) (4 females and 6 males) were included. Detailed information of brain tissue is provided in Table 1. Each frozen brain sample was weighed and homogenized in 10X volume of radio-immunoprecipitation assay (RIPA) lysis buffer (0.5 M Tris-HCl, pH 7.4, 1.5 M NaCl, 2.5% deoxycholic acid, 10% NP-40, 10 mM EDTA, 20–188) containing 1 mM phenylmethylsulfonyl fluoride (PMSF), protease inhibitor cocktail, and halt phosphatase inhibitor cocktail, followed by sonication and centrifugation for 15 min at 16,000×g at 4 °C to remove cellular debris. Supernatants were collected, protein concentration was determined by Bradford assay, and samples were processed for immunoblotting.

**Table 1:** Human brain samples

Case	Pathology Dx	Thal	Braak	Clinical Dx	Age at Death	Sex
1	Normal		2	aMCI	70	Male
2	Normal		0	Normal	56	Female
3	Normal		0	Normal	57	Female
4	Normal	0	2	AD v DLB	69	Male
5	Normal	1	1	DA	64	Female
6	Normal	0	3	DLB v FTD	63	Male
7	Normal		0	Normal	61	Female
8	Normal	1	1	NAIM	60	Female
9	Normal	0	1	PSP/PLS	56	Female
10	Normal	0	1	TD	61	Male
1	DLBD	0	0	DLB	60	Male
2	DLBD	0	2	DLB	61	Male
3	DLBD	0	2	PDD	66	Male
4	DLBD	0	0	PDD	68	Female
5	DLBD	1	2	PSP	72	Female
6	DLBD	1	2	DLB (RBD)	70	Male
7	DLBD	1	2	PDD	56	Male
8	DLBD	1	2.5	PDD	62	Female
9	DLBD	0	2	PD-MCI	66	Male
10	DLBD	2	1	PDD v CBD	69	Female

*Dx* Diagnosis, *AD* Alzheimer’s diseases, *aMCI* Amnestic mild cognitive impairment, *CBD* Corticobasal degeneration, *DA* Dysautonomia, *DLBD* Diffuse lewy body disease, *DLB* Dementia with lewy bodies, *FTD* Frontotemporal dementia, *NAIM* Nonvasculitic autoimmune inflammatory meningoencephalitis, *PD* Parkinson’s disease, *PDD* Parkinson’s disease with dementia, *PLS* Primary lateral sclerosis, *PSP* Progressive supranuclear palsy, *RBD* REM sleep behavior disorder, *TD* Torsion dystonia

### Immunofluorescence

Cells were cultured on 12-mm glass coverslips with or without 1 μg/ml tetracycline for 72 h. Cells were washed with phosphate-buffered saline (PBS) and incubated with 300 nM with MitoTracker-Green (Molecular Probes, Inc., Eugene, OR, USA) according to the manufacturer’s protocol to visualize mitochondria. Cells were fixed with 4% paraformaldehyde for 10 min at room temperature (RT) and washed three times in 1X Tris-buffered saline (TBS) (500 mM NaCl, 20 mM Tris, pH 7.4), blocked for 1 h in 1.5% goat serum, 0.5% Triton X-100 in 1X TBS and incubated overnight at 4 °C with primary antibodies (SIRT3 and human αsyn). The following day cells were washed and treated with Alexa Fluor® 488 and 568 secondary antibodies for 1 h at RT (see Table 2, for details of the antibodies used in the study). Coverslips were mounted on Super Frost Plus slides with Vectashield Hardset (Vector Labs, Burlingame, CA) and cells were visualized using an Axio observer inverted microscope (Carl Zeiss, Germany).

**Table 2:** Antibodies used for western blot and immunocyhistochemistry

Antibody	Source	Dilution
α-Synuclein (mouse)	BD Transduction Laboratories (61078)	1:2000 (WB)
α-Synuclein (mouse)	Biolegend (SIG39730)	1:2000 (WB, ICC)
α-Synuclein, clone 5G4 (mouse)	Millipore (MABN389)	1:1000 (WB)
Oligomer A11 (rabbit)	Thermo Fisher Scientific (AHB0052)	1:1000 (WB)
SIRT3 (mouse)	Santa Cruz (sc-135,796)	1:1000 (WB)
SIRT3 (rabbit)	Cell Signaling (2627 s)	1:2000 (WB)
SIRT3 (rabbit)	Novus Biologicals (NBP1–31029)	1:1000 (WB)
		1:500 (ICC)
SIRT3 (rabbit)	Cell Signaling (5490)	1:1000 (WB)
Heme oxygenase-1 (rabbit)	Cell Signaling (5853 s)	1:1000 (WB)
AMPK (rabbit)	Cell Signaling (5831 T)	1:1000 (WB)
Phospho-AMPK (rabbit)	Cell Signaling (2535 s)	1:1000 (WB)
CREB (rabbit)	Cell Signaling (4820 s)	1:1000 (WB)
Phospho-CREB (rabbit)	EMD Millipore (06–519)	1:2000 (WB)
DRP1 (rabbit)	Bethyl Laboratories (A303-410A-M)	1:2000 (WB)
Phospho-DRP1 (rabbit)	Cell Signaling (3455 s)	1:1000 (WB)
SOD2 (rabbit)	abcam (ab13533)	1:5000 (WB)
SOD2 (acetyl K68) (rabbit)	abcam (ab137037)	1:2000 (WB)
COX IV (rabbit)	Cell Signaling (4850 s)	1:1000 (WB)
GAPDH (rabbit)	Santa Cruz (sc-25,778)	1:4000 (WB)
	Abgent (AP7873a)	1:4000 (WB)
Actin (mouse)	Sigma (A5316)	1:7500 (WB)
GM130 (rabbit)	abcam (ab52649)	1:2000 (WB)
Alexa Fluor 488 (goat anti-mouse)	Thermo Fisher Scientific (A11001)	1:500 (ICC)
Alexa Fluor 568 (goat anti-rabbit)	Thermo Fisher Scientific (A11011)	1:500 (ICC)
Goat anti-mouse HRP	Southern biotech (1010–05)	1:5000 (WB)
Goat anti-rabbit HRP	Southern biotech (4010–05)	1:5000 (WB)
IRDye® 680RD Goat anti-Mouse IgG (H + L)	LI-COR® (926–68,070)	1:10000 (WB)
IRDye® 800CW Goat anti-Mouse IgG (H + L), 0.5 mg	LI-COR® (926–32,210)	1:10000 (WB)
IRDye® 800CW Goat anti-Rabbit IgG (H + L), 0.5 mg	LI-COR® (926–32,211)	1:10000 (WB)

*WB* Western blot, *ICC* Immunocytochemistry

### Gaussia luciferase protein-fragment complementation assays

Luciferase activity was measured in 15μg cell lysate or in freshly homogenized STR and SN rat tissue in multilabel plate reader at 480 nm (EnVision, PerkinElmer; Waltham, MA, USA) following the injection of the substrate, coelenterazine (40 μM, NanoLight tech, AZ, USA) with a signal integration of 2 s.

### Western blotting analysis

To prepare whole cell lysates, cells were washed twice with ice-cold PBS and total proteins were isolated by incubating H4 cells in RIPA lysis buffer (50 mM Tris–HCl, pH 7.4, 150 mM NaCl, 1 mM EDTA, 1 mM EGTA, 1.2% Triton X-100, 0.5% sodium deoxycholate, and 0.1% SDS, ADI-80-1496,1 mM PMSF) or primary neurons in a triton-X based lysis buffer (150 mM NaCl, 1 mM EDTA, 20 mM Tris-HCL,1% triton-X pH 7.4). Both buffers were supplemented with protease inhibitor cocktail, and halt phosphatase inhibitor cocktail. Collected cells were centrifuged at 10,000×g for 10 min at 4 °C. The protein concentration was determined with Bradford reagent. 15 μg proteins were separated on Bis-Tris polyacrylamide gradient gels (NuPAGE Novex 4–12% Bis-Tris Gel, Life tech) and transferred to nitrocellulose membranes. Membranes were then blocked for 1 h at RT in TBS-T (500 mM NaCl, 20 mM Tris, 0.1% Tween 20, pH 7.4) supplemented with 10% non-fat dried milk. Subsequently membranes were incubated overnight at 4 °C with primary antibodies followed by 1 h at RT with HRP-conjugated secondary antibodies or IRDye® conjugated secondaries (LI-COR®) (Table 2). Proteins were detected using an enhanced chemiluminescent detection system (ECL, EMD Millipore) and a CCD imaging system (LAS-4000, Fujifilm, Japan) or Odyssey® CLx Imaging System (LI-COR®, USA).

### Mitochondria/cytosol fractionation

Cells were homogenized in buffer A (0.25 M sucrose, 10 mM Tris–HCl [pH 7.5], 10 mM KCl, 1.5 mM MgCl_2_, 1 mM EDTA, 1 mM dithiothreitol, and 0.1 mM PMSF). Homogenates were centrifuged at 700×g for 5 min at 4 °C, and supernatants were collected and centrifuged at 10,000×g for 30 min at 4 °C. The supernatant was designated the cytosolic fraction, and the pellet was used as the mitochondrial enriched fraction. The pellets were resuspended in buffer B (0.25 M sucrose, 10 mM Tris–HCl [pH 7.5], 10 mM KCl, 1.5 mM MgCl_2_, 1 mM EDTA, 1 mM dithiothreitol, 0.1 mM PMSF, and 1% NP 40). To confirm the purity of the mitochondrial fraction, the lysates were probed for the specific mitochondria marker cytochrome *c* oxidase IV (COXIV).

### Dot blot assay

Tris-buffered saline (TBS)-wetted nitrocellulose membrane (NC membrane, 0.45 μm pore) was mounted on the Bio-Dot microfiltration apparatus (cat. no. 1706545, Bio-Rad). 15 μg proteins were loaded into the wells of a microfiltration apparatus under mild vacuum. After washing with TBS, the NC membranes were blocked with TBS-T (500 mM NaCl, 20 mM Tris, 0.1% Tween 20, pH 7.4) supplemented with 10% non-fat dried milk and incubated overnight at 4 °C with primary antibodies (anti-oligomer A11, cat. no. AHB0052; anti-αsyn 5G4, cat. no. MABN389 followed by 1 h at RT with HRP-conjugated secondary antibodies. Proteins were detected using an enhanced chemiluminescent detection system (ECL, EMD Millipore) and ChemiDoc MP Imaging System (Bio-Rad, 170–01402, USA).

### Isolation of rat brain mitochondria

SN were dissected and homogenized in 0.5 mL of ice-cold MIBA (10 mM Tris–HCl [pH 7.4], 1 mM EDTA, 0.2 M D-mannitol, 0.05 M sucrose, 0.5 mM sodium orthovanadate, 1 mM sodium fluoride and dissolved in water) containing 1X protease inhibitors with a hand-held homogenizer for 40 strokes on ice. The homogenate was transferred into 1.5 mL tubes and then centrifuged at 500×g for 5 min. The pellet was discarded, and remaining supernatant was centrifuged at 11,000×g for 20 min at 4 °C, yielding the heavy mitochondrial (HM, pellet) and the light mitochondrial (LM, supernatant) fraction. The HM pellet was washed twice with 1 mL ice-cold MIBA buffer it was resuspended in 0.1–0.3 mL of MIBA to yield the final solution enriched in mitochondria.

### Mitochondrial respiration analysis

The oxygen consumption rate (OCR) was assessed using a Seahorse Bioscience XF96 analyzer (Seahorse Bioscience, Billerica, MA, USA) in combination with the Seahorse Bioscience XF Cell Mito Stress Test assay kit according to the manufacturer’s recommendations. H4 SL1&SL2 cells were seeded in 12-wells of a XF 96-well cell culture microplate (Seahorse Bioscience, 102,601–100) and grown to 70% confluency in 200 μL of growth medium prior to analysis. On the day of assay, culture media were changed to assay medium with 175 μL (Dulbecco’s Modified Eagle’s Medium, D5030), supplemented with 25 mM glucose, 2 mM glutamine, and 2 mM pyruvate. Prior to assay, plates were incubated at 37 °C for 1 h without CO_2_. Thereafter successive OCR measurements were performed consisting of basal OCR, followed by OCR level after the automated injection of 25 μl oligomycin (20 μM), 25 μl carbonyl cyanide 4-(trifluoromethoxy) phenylhydrazone (FCCP) (20 μM), and a combination of 25 μl rotenone + antimycin A (12 μM), respectively. After the assays, plates were saved and OCR was normalized to the total protein amount per well.

### SIRT3 siRNA transfection

Small interfering RNAs (siRNAs) for human SIRT3 (sc-61,555, Santa Cruz Biotechnology, CA, USA) and control non-target siRNA (SN-1003, Negative Control, Bioneer, Daejeon, Korea) were reconstituted in siRNA buffer (Qiagen, CA) following the manufacturer’s instructions and transfections of conducted using Lipofectamine 2000 reagent (Invitrogen, CA, USA). Briefly H4 SL1&SL2 cells were seeded in 60-mm culture dishes for 24 h before transfection. Subconfluent cells were treated either with SIRT3 siRNA (100 nM) or non-targeting siRNA (20 nM) complexed with lipofectamine for 2 h. The extent of knockdown was evaluated by western blot analysis.

### Determination of mitochondrial ROS

MitoSOX™ Red fluorescent probe (Molecular Probes, Inc., Eugene, OR, USA) was used to visualize mitochondrial superoxide production according to the manufacturer’s protocol. Briefly, H4 SL1&SL2 grown on 12-mm glass were washed twice with PBS to remove the medium and incubated with 2.5 μM MitoSOX-Red reagent in the dark at 37 °C. Cells were washed gently three times with warm PBS buffer and imaged immediately after, under fluorescence microscopy. To confirm mitochondrial localization of MitoSOX-Red, cells were loaded with 300 nM MitoTracker-Green (Molecular Probes, Inc., Eugene, OR, USA) for 30 min. The mean fluorescence intensities of MitoSOX-Red and MitoTracker-Green were divided by the number of cells in each image and quantified using Image J software.

### Statistical analysis

All data were analyzed by the Graph Pad Prism 7 software (San Diego, CA) and statistical significance was determined by one-way ANOVA analysis of variance with Tukey’s multiple comparisons test. Results presented as mean ± standard deviation (SD). For isolated mitochondria studies in vivo and LBD brain, a Mann-Whitney U test was used to analyze the Western blots, Differences were considered to be statistically significant with *p < 0.05, **p < 0.01.

## Results

### Increased αsyn oligomers correlate with decreased mitochondrial SIRT3 protein levels

**Fig. 1:**
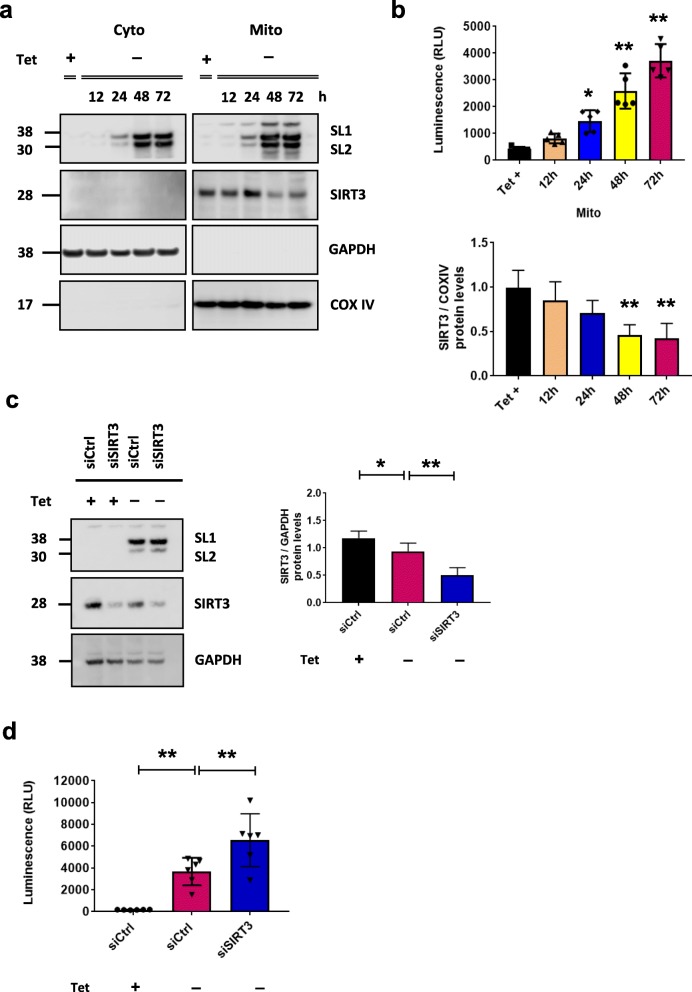
αSyn is found in mitochondria-enriched fraction of H4 SL1&SL2 cells and induces a decrease in SIRT3 expression. **a** Representative cropped western blots showing αsyn and SIRT3 in cytosolic and mitochondrial fractions at different time points (0 – 72 h) (**b**) αsyn oligomers are apparent after 24 h by luciferase assay (RLU: relative luminescence units), *n* = 5. Quantification of SIRT3 protein level in mitochondria demonstrates significant decrease in SIRT3 at 48 h and 72 h. **c** Whole cells lysates from H4 SL1&SL2 cells demonstrate decreased SIRT3 expression after transfection with SIRT3 siRNA *n* = 6 (**d**) Luciferase activity from αsyn oligonmerization is significantly increased in cells transfected with SIRT3 siRNA (siSIRT3) compared to control siRNA (siCtrl). Error bars represent the mean ± SD. *p < 0.05, **p < 0.01. Note: In (**a**) αsyn and SIRT3 bands are from different experiments run on different gels. COXIV, GAPDH, and SIRT3 are all from same samples and immunoblot. Loading controls for αsyn blot are not shown. In panel (**c**) αsyn, SIRT3, and GAPDH are detected on same immunoblot

Although it has been described previously that αsyn localizes to mitochondria and αsyn overexpressing cells exhibit mitochondrial dysfunction [1, 27, 28] the relationship between αsyn and mitochondria in pathologic conditions and the mechanisms whereby αsyn may induce mitochondrial dysfunction are still poorly understood. Herein, we use a previously described inducible cell model of human αsyn overexpression that results in formation of intracellular oligomeric species over time [24]. This tetracycline-off (Tet-off) stable cell line facilitates monitoring of αsyn oligomerization in situ via a split luciferase protein–fragment complementation assay. To determine if oligomeric αsyn species associate with mitochondria, cells were harvested at various time points after tetracycline removal, mitochondrial-enriched fractions were isolated (Fig. 1a), and luciferase activity was measured as a surrogate for αsyn oligomeric species. Luciferase activity increased in a time-dependent manner in both the mitochondrial (Fig. 1b) and cytosolic fractions (Additional file 1: Figure S1a) of the cells. The presence of αsyn oligomers in mitochondrial-enriched fractions was confirmed by the detection of increased high molecular weight species on a native western blot 72 h after removal of tetracycline (Additional file 1: Figure S1b) and on a dot blot, using the amyloid-specific antibody A11 [29] and αsyn disease-associated 5G4 antibody [30] (Additional file 1: Figure S1c). The purity of mitochondrial-enriched fractions was confirmed by western blotting for the presence of mitochondrial inner membrane specific protein COXIV, and the absence of cytosolic GAPDH and Golgi marker (GM130) (Additional file 1: Figure S1b). Interestingly, the increase in mitochondrial-associated αsyn oligomers was accompanied by a decrease in SIRT3 protein levels beginning 12 h after αsyn expression was turned on, and becoming significant by 48 h (Fig. 1a - b). Immunocytochemistry confirmed decreased SIRT3 immunofluorescence in cells accumulating αsyn oligomers (Additional file 1: Figure S1d, Tet– 72 h) compared to control (Additional file 1: Figure S1c, Tet + 72 h). To confirm that the decrease in SIRT3 is directly related to αsyn oligomers we used siRNA to knock-down SIRT3 expression and observed a reciprocal increase in αsyn oligomerization in our cellular model (Fig. 1c - d, **p < 0.01). To confirm that association of αsyn with mitochondria is not influence by the split luciferase tags, H4 cells expressing wild-type, untagged αsyn were fractionated and αsyn was detected in mitochondria-enriched fraction of H4 WT-αsyn overexpressing cells (Additional file 2: Figure S2a). Furthermore, transduction of 7 days in vitro (DIV) mouse primary cortical neurons with AAV-expressing untagged human wt-αsyn revealed a significant, albeit modest, decrease of SIRT3 in total cell lysates from neurons overexpressing αsyn (Additional file 2: Figure S2b, **p < 0.01) when compared to control transduced neurons (AAV-Venus YFP).

### Alpha-synuclein associated decrease in SIRT3 is via AMPKα-CREB signaling pathway

**Fig. 2:**
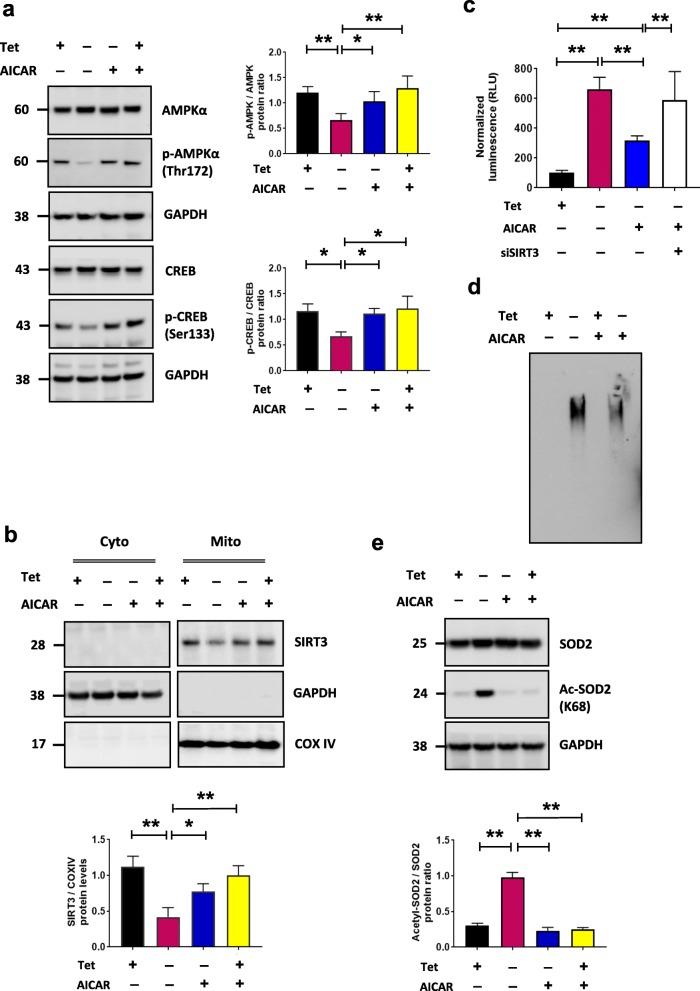
AICAR activates AMPK-CREB signaling pathway and increases SIRT3 activity and reduces αsyn oligomers. **a** Representative cropped western blots showing AMPKα, p-AMPKα (Thr 172), CREB, and p-CREB (Ser 133) in H4 SL1&SL2 cells with/without 2 mM AICAR. **b** SIRT3 expression increases with AICAR-treatment, *n* = 3. **c** Luciferase assay (*n* = 5) demonstrates AICAR significantly decreases αsyn oligomers and knockdown of SIRT3 (siSIRT3) prevents reduction of αsyn oligomers by AICAR. **d** Native-page shows AICAR significantly decreases αsyn oligomers. **e** Representative cropped western blot showing increased Ac-SOD2 (acetyl K68) with no change in total SOD2 in whole cells lysates. AICAR restored acetylated SOD2 levels, *n* = 3. Error bars represent the mean ± SD, (*n* = 3–5). *p < 0.05, **p < 0.01. In panel (**a**) the same samples were run on different gels and probed separately for AMPKα, p-AMPKα, and GAPDH, and CREB, p-CREB, and GAPDH respectively. In panels (**b**) and (**e**) separate blots were probed for SIRT3 and GAPDH, and COXIV, or SOD2, Ac-SOD2, and GAPDH

Cells with decreased SIRT3 function have been shown to have reduced phosphorylation of AMPKα [14, 31, 32] and cAMP response element binding protein (CREB). In addition, previous studies have shown that overexpression of αsyn can reduce AMPKα activation in neuronal cells [33]. Because expression of αsyn results in decreased SIRT3 expression, we next examined the levels of phosphorylated-AMPKα (pAMPKα) and phosphorylated CREB (p-CREB). At 72 h, when SIRT3 expression is significantly decreased and αsyn is present in the mitochondrial fraction (Fig. 1a), we detected a significant decrease in p-AMPKα (Thr172) and p-CREB (Ser133) (Fig. 2a). To further validate modulation of the AMPKα-CREB signaling pathway by αsyn oligomers we asked whether treatment with 5-aminoimidazole-4-carboxamide-1-β-d-ribofuranoside (AICAR), an AMPKα agonist, can rescue αsyn-induced changes in mitochondrial SIRT3 and associated signaling proteins. Following treatment with 2 mM AICAR [34], a significant increase in p-AMPKα and p-CREB levels was observed after 72 h (Fig. 2a, *p < 0.05) and importantly, a significant restoration of SIRT3 levels (Fig. 2b, *p < 0.05) accompanied by a significant decrease in αsyn oligomers observed by luciferase assay (Fig. 2c) and detected by native PAGE as well (Fig. 2d). AICAR failed to reduce αsyn oligomers when SIRT3 expression is knocked down using siRNA, indicating that the effect of AICAR treatment is dependent on the presence of SIRT3 (Fig. 2c). Because SIRT3 is a major mitochondrial deacetylase, we examined the acetylation state of a known substrate, superoxide dismutase 2 (SOD2) [35], in cells overexpressing αsyn oligomers. Acetylated SOD2 (K68) is significantly increased in cells overexpressing αsyn compared to control (Fig. 2e, **p < 0.01), consistent with reduced SIRT3 activity, and AICAR treatment decreases the acetylation status, consistent with a restoration of SIRT3 levels. Of note, αsyn overexpression had no effect on total SOD2 levels which remained consistent in all conditions (Fig. 2e).

### SIRT 3 activation attenuates αsyn-induced mitochondrial ROS

**Fig. 3:**
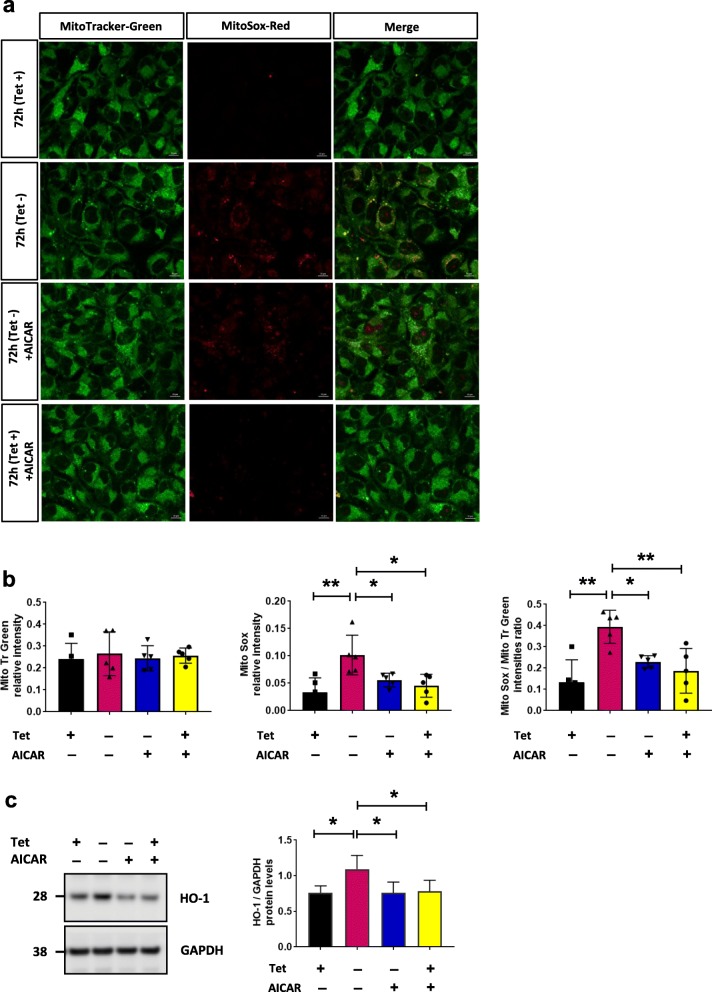
Activation of SIRT3 by AICAR attenuates ROS production. **a** Fluorescence microscopy images of MitoSox-Red and Mitotracker-Green staining in fixed H4 SL1&SL2 cells. MitoSox-Red fluorescence increases when αsyn is overexpressed and AICAR treatment attenuates mtROS. Representative images from 3 experiments. MitoTracker-Green (mitochondria; green); MitoSox-Red (mitochondria; red); merged images (yellow). Scale bar = 10 μm. **b** Relative intensity of MitoTracker-Green signal between conditions. Quantification of MitoSOX-Red signal intensities (*n* = 5), and mean intensity of MitoSOX-Red normalized to MitoTracker-Green intensity (**c**) Representative cropped western blot of HO-1 and GAPDH in whole cells lysates from H4 SL1&SL2 cells. HO-1 level increases at 72 h and is reduced after AICAR-treatment, *n* = 5. Error bars represent the mean ± SD. *p < 0.05, **p < 0.01

Because SIRT3 plays a crucial role in modulating reactive oxygen species (ROS) and limiting oxidative damage of cellular components [36], we asked whether the presence of αsyn in mitochondrial fraction is associated with increased oxidative stress that can be rescued with SIRT3 activation. Cells overexpressing αsyn were stained with Mitotracker-Green, to visualize mitochondria and control for total levels of mitochondria, and MitoSOX-Red to monitor mitochondrial ROS production. Fluorescence microscopy revealed increased ROS at 72 h compared to control conditions (Tet + 72 h) (Fig. 3a - b), and as predicted, AICAR-treatment reduced ROS production (Fig. 3a and b, bottom row). Of note, neither expression of αsyn nor AICAR treatment altered the total number of mitochondria (Fig. 3b). Increased oxidative stress and ROS can induce the expression of heme oxygenase-1 (HO-1) [37] and increased HO-1 mRNA and protein expression have been reported in a wide spectrum of diseases including neurodegenerative diseases such as Parkinson disease [38, 39]. In line with these data, we found a significant increase of HO-1 in cells expressing αsyn for 72 h (Fig. 3c), and a concomitant decrease of HO-1 in cells treated with AICAR compared to control (Tet + 72 h) (Fig. 3c).

### αSyn impairs mitochondrial dynamics and bioenergetics which can be rescued by activation of SIRT3

**Fig. 4:**
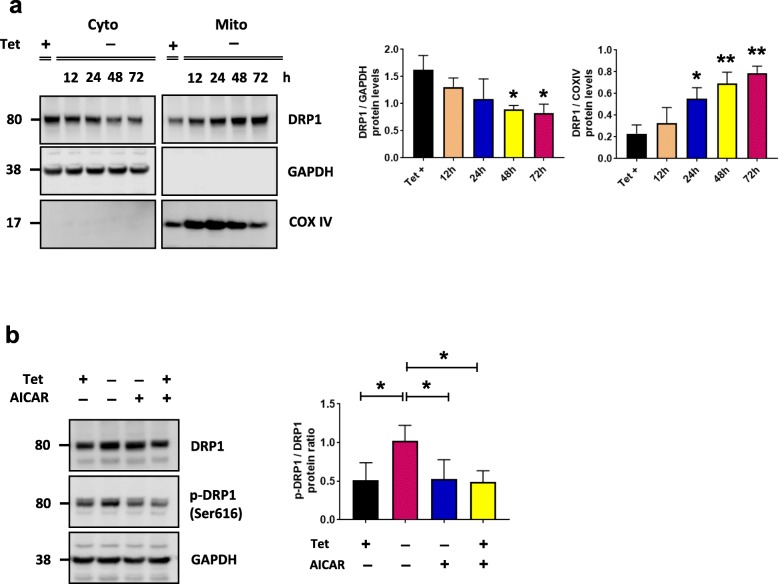
αSyn expression affects mitochondrial dynamics and can be rescued with AICAR. a Representative cropped western blot showing DRP1 in cytosol and mitochondria from H4 SL1&SL2 cells over time (0 – 72 h). Quantitation of protein levels for DRP1 in cytosol and mitochondria (*n* = 3). DRP1 band was normalized to respective loading controls GAPDH and COXIV. **b** Representative cropped western blot from showing DRP1and p-DRP1 levels in cells treated with or without AICAR (*n* = 4). Error bars represent the mean ± SD. *p < 0.05, **p < 0.01

**Fig. 5:**
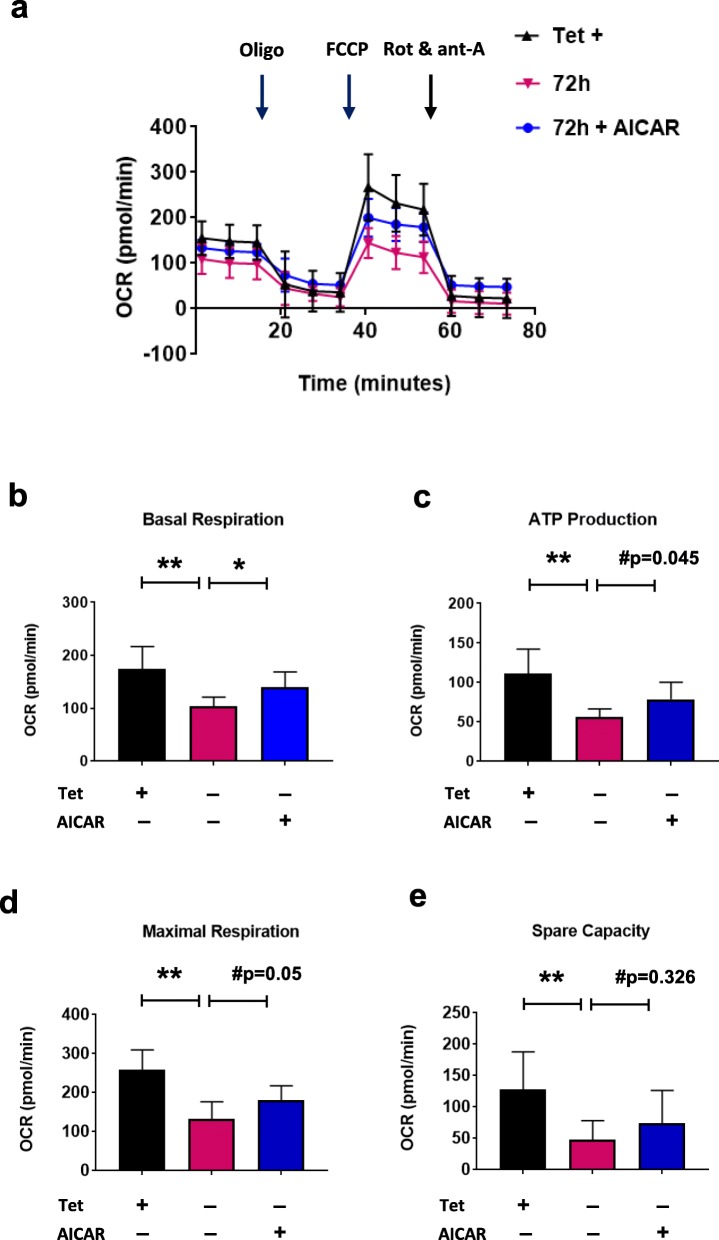
AICAR rescues basal respiration deficit induced by αsyn oligomers. **a** Mitochondrial OCR was assessed by Seahorse XFe96 Analyzer. The OCR is significantly reduced in cells overexpressing αsyn and AICAR significantly improves basal respiration (**b**)**.** ATP production maximal respiration are trending towards rescue with AICAR (**c**, **d**) but AICAR had no effect on spare capacity (**e**). Error bars represent the mean ± SD. *p < 0.05, **p < 0.01. Note: #p is *p* value obtained with student t-test as one-way ANOVA analysis did not quite reach significance of *p* < 0.05

Mitochondrial dynamics play a critical role in maintaining mitochondrial health, and are thus crucial for neuronal function and survival. Changes in the expression and/or localization of fission/fusion proteins can impair this process and induce cell death. To determine the effect of mitochondrial-associated αsyn on mitochondrial dynamics, we examined the expression of DRP1. In the presence of αsyn, we observed a recruitment of DRP1 from the cytosol to the mitochondria (Fig. 4a) as well as an increase in the phosphorylated form of DRP1 at serine 616 (p-DRP1) (Fig. 4b, Additional file 3: Figure S3). Both of these events suggest an activation of mitochondrial fission in our cellular model that may lead to mitochondrial fragmentation [40–42]. When we evaluated the effect of AICAR on mitochondria dynamics we found a significant decrease in p-DRP1 (Fig. 4b). To determine if αsyn associated with the mitochondrial fraction affects cellular bioenergetics, we measured the OCR in live cells using the Seahorse XF96 analyzer. The OCR was measured under basal conditions followed by the sequential addition of oligomycin (ATP synthase inhibitor), carbonyl cyanide 4-(trifluoromethoxy) phenylhydrazone (FCCP; mitochondrial uncoupler), and rotenone plus antimycin A (Complex I and III inhibitor) to assess ATP production, maximal respiration, and spare capacity respectively. Cells overexpressing αsyn had significantly decreased OCR in all paradigms tested when compared to control cells (Tet+) (Fig. 5a - e). This is highly suggestive of a mitochondria respiratory deficit in the presence of mitochondrial αsyn. AICAR treatment was able to significantly restore the OCR level of basal respiration (Fig. 5b, *p < 0.05). Although restoration of ATP production and maximal respiration did not quite reach significance, a trend was observed (Fig. 5c - d). Taken together, our data support a hypothesis whereby increased αsyn results in decreased mitochondrial function via a SIRT3-dependent cascade of events that can be rescued by increasing SIRT3 levels using an AMPKα agonist.

### Decreased SIRT3 is also detected in vivo in rodents overexpressing alpha-synuclein

**Fig. 6:**
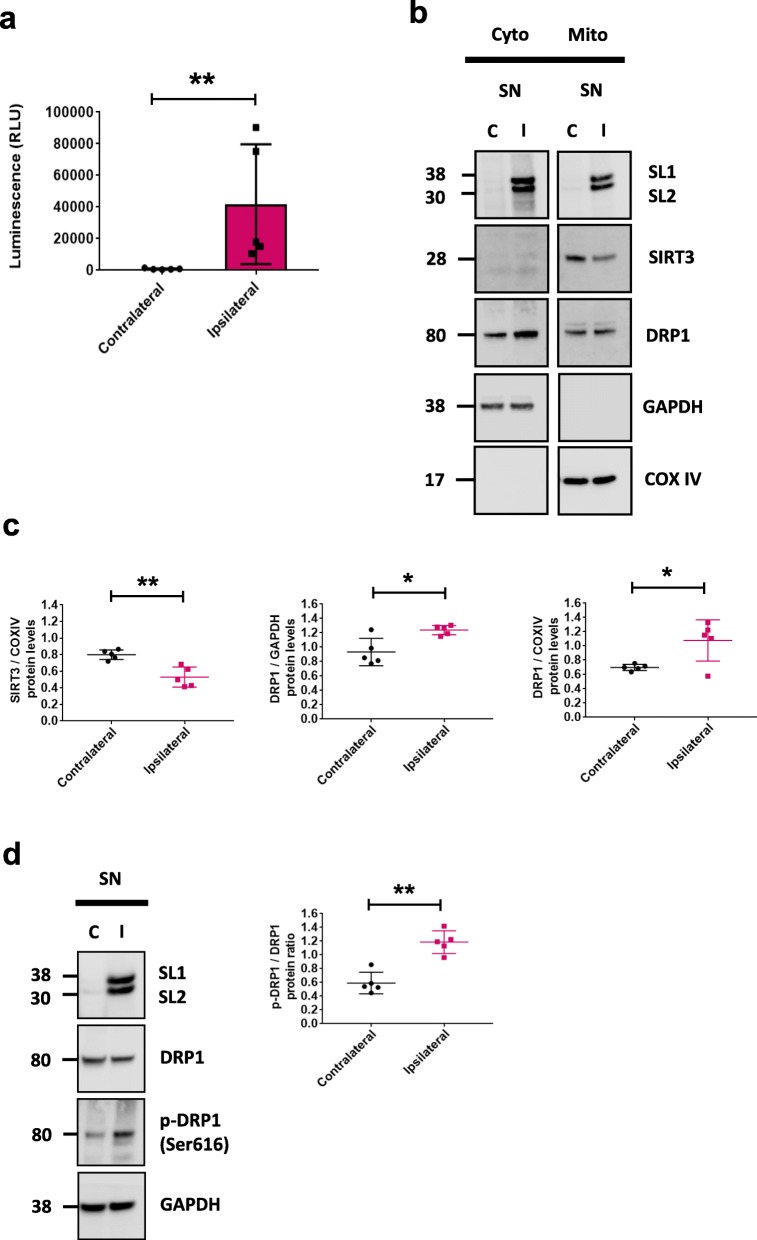
αSyn expression decreases SIRT3 and alters mitochondrial dynamics in vivo. **a** αSyn oligomers were quantified via luciferase assay in rat brain homogenates at 4 weeks after stereotaxic injection of AAV8-SL1&SL2 in SN, *n* = 5. **b** Representative cropped western blots showing αsyn, SIRT3, DRP1 in cytosol and mitochondria from SN of rats 4 weeks after stereotaxic injection of AAV8-SL1 and AAV8-SL2. (C). **c** Quantification of αsyn, SIRT3, DRP1, protein levels in cytosol and mitochondria from two separate blots for each of 4–5 rats. All bands were normalized to respective loading controls GAPDH and COXIV. d Representative cropped western blot of αsyn, DRP1, and p-DRP1 (Ser 616) in SN lysate of AAV8-SL1&SL2 injected rat. αSyn expression leads to increased p-DRP1 protein levels in ipsilateral (I) injected SN compared to contralateral (C) uninjected SN, *n* = 5 rats total. In panel (**a**) the same samples were run on one blot that was cropped prior to immunoblotting for αsyn, SIRT3, COXIV, DRP1, and GAPDH. Error bars represent the mean ± SD, (*n* = 4–5 rats). *p < 0.05, **p < 0.01

Although a recent study demonstrated that overexpression of SIRT3 in a rodent model of αsyn overexpression could rescue αsyn-induced cell loss in the SN pars compacta [11], the mechanism by which SIRT3 exerts its neuroprotective effects was not addressed. To confirm the findings of the previous study and determine if similar mechanisms are at play in vivo to those described in our cellular studies, we used a rodent model whereby accumulation of αsyn oligomers in rat SN after 4 weeks is accompanied by significant loss of dopaminergic neurons. This animal model aligns with the cellular model used for the majority of experiments described herein where injection of AAV2/8-human split-luciferase αsyn facilitates monitoring of αsyn oligomerization in situ via a protein–fragment complementation assay. We have previously shown that unilateral injection into SN of adult rat results in abundant expression of αsyn oligomeric species in both cell bodies and axon terminals of the nigrostriatal pathway [25]. Here, at 4 weeks post viral injection we performed a luciferase assay on fresh homogenate of dissected SN to confirm the presence of αsyn oligomers (Fig. 6a, **p < 0.01). Subsequent subcellular fractionation of nigral tissue was performed to assess SIRT3 levels in mitochondria in the ipsilateral (injected) side of the rat brain. Consistent with our in vitro data, accumulation of αsyn in SN was accompanied by a significant decrease in SIRT3 (Fig. 6b - c, **p < 0.01, *p < 0.05). Of note there was no difference in SIRT3 levels in SN in control animals that received an injection of AAV8 expressing *gaussia* luciferase only (Additional file 4: Figure S4a). Surprisingly, despite a decrease of SIRT3 level, we did not observed a decrease of cytosolic DRP1 as previously described but rather a significant increase (Fig. 6c). However, examination of DRP1 levels in our rodent model revealed a significant increase of p-DRP1 in the injected SN (Fig. 6d, **p < 0.01), mimicking once again our in vitro observation. Lastly, AMPKα-CREB signaling was downregulated in the SN of these animals (Additional file 4: Figure S4b).

### SIRT3 levels are decreased in human Lewy body disease brains

**Fig. 7:**
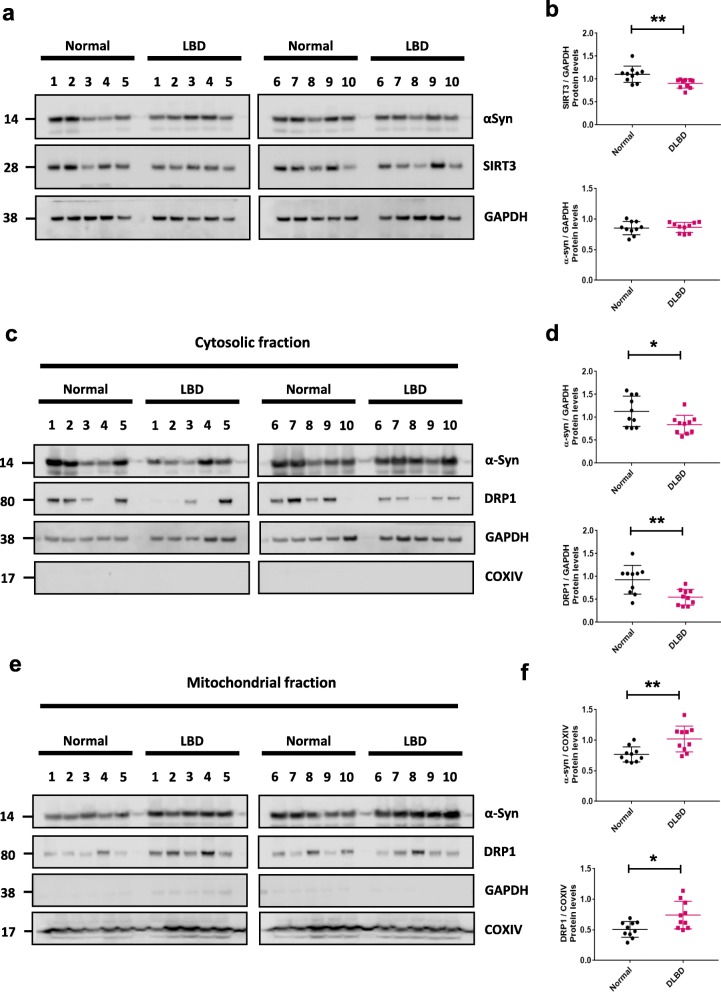
SIRT3 is decreased in human post-mortem brain of neuropathologically confirmed Lewy body disease individuals. **a** Representative cropped western blot from *n* = 3 showing decreased SIRT3 in total brain lysates from ten LBD brains compared to ten age-matched healthy controls. **b** Quantification of αsyn, SIRT3 protein levels from *n* = 3 western blots. **c**, **d** Representative western blot of cytosolic fraction. DRP1 and αsyn were quantified using GAPDH as a loading control. **e**, **f** Representative western blot of mitochondrial fraction. DRP1 and αsyn were quantified using COXIV as a loading control. Error bars represent the mean ± SD. **p < 0.01

Lastly, we assessed the level of SIRT3 in human post mortem brain with a confirmed neuropathological diagnosis of Lewy body disease (LBD) (Table 1). Frozen striatal tissue from 10 LBD and 10 age-matched healthy controls were homogenized, run on SDS-PAGE, and probed with antibodies to detect αsyn, SIRT3, and DRP1. Western blot analyses showed significantly reduced expression of SIRT3 in LBD brains compared to controls but no significant difference in the level of αsyn and DRP1 compared to controls (Fig. 7a - b). Brains from both sexes were utilized but there was no difference in the interpretation of the data when stratified by sex (data not shown). Interestingly, subcellular fractionation of LBD brain homogenates also revealed a decrease in cytosolic levels of DRP1 and αsyn in LBD samples (Fig. 7c - d), with a corresponding increase in levels of mitochondrial localized DRP1 and αsyn (Fig. 7e - f) when compared to controls. Together, these results are consistent with our findings from cell and rodent models where decreased SIRT3 protein levels were observed when αsyn localizes to the mitochondria.

## Discussion

**Fig. 8:**
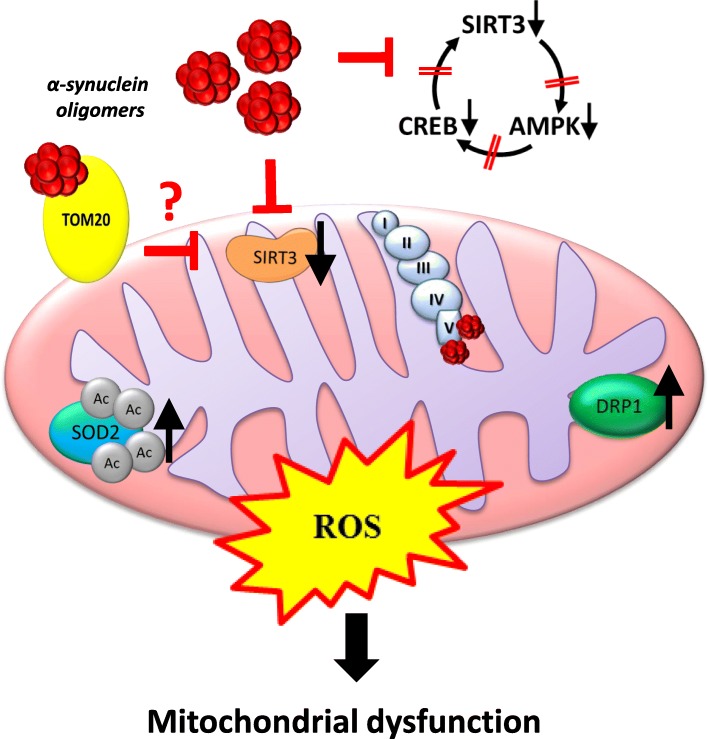
Cartoon representing of αsyn-induced effect on SIRT3 and mitochondrial dysfunction. Consequences of decreased SIRT3 include decreased AMPK-CREB signaling, impairment in mitochondrial bioenergetics and dynamics, and increased acetylation of SIRT3 substrates such as SOD2 all of which contribute to increased ROS production and neurodegeneration. Question mark indicates pathway not supported by data in this manuscript

Herein, we identify a cellular mechanism that illuminates how αsyn-associated mitochondria may lead to mitochondrial dysfunction and the initiation of a self-perpetuating cycle of aggregation, deficient cellular metabolism, and eventually cell death (Fig. 8). For the first time we identify αsyn oligomers in the mitochondrial enriched fraction of cells and a consequent decrease in SIRT3 activity in multiple model systems including cell culture, rodent models, and human post-mortem brains with a neuropathological diagnosis of LBD. We demonstrate that the presence of αsyn oligomers in mitochondrial-enriched fractions correlates with decreased mitochondrial function and decreased SIRT3 expression and function. Interestingly, we show that SIRT3 downregulation is accompanied by dysregulation of the AMPK signaling pathway, perturbation of fission mechanisms, and impairment of basal respiration, all of which contribute to increased ROS and mitochondrial dysfunction. These findings are observed not only in an experimental cellular model, but also a rodent model of αsyn accumulation, and importantly, in human post mortem LBD brain. Lastly, treatment with an AMPK agonist, AICAR, appears to improve αsyn-associated mitochondrial dysfunction by decreasing αsyn oligomer formation and increasing SIRT3 expression. Overall, these results confirm the health enhancing capabilities of SIRT3 and validate its potential as a new therapeutic target for PD and related disorders.

Mitochondrial dysfunction has been linked to the pathogenesis of neurodegenerative diseases including PD, with mutations identified in mitochondrial-associated proteins such as PINK1 and parkin causing familial PD [43, 44]. αSyn, a major neuropathological hallmark of PD and alpha-synucleinopathies can perturb mitochondria and previous studies have shown that overexpression of αsyn has dramatic effects on mitochondrial morphology, reduces respiratory chain complex activity, and impairs mitochondrial functions in vitro and in vivo [45, 46]. Accumulation of wild-type αsyn and truncated species within the mitochondria has been described [47, 48] however, no study has directly demonstrated that oligomeric αsyn species can associate mitochondria. Here, we used a split luciferase protein complementation assay to demonstrate accumulation of αsyn oligomers in the mitochondrial-enriched fraction of cells in culture and rat brain homogenates. We speculate that the presence of oligomeric αsyn species triggers a cascade of events leading to mitochondria malfunction associated with PD pathogenesis. However, our data do not preclude the possibility that cytosolic αsyn may contribute to the observed mitochondrial dysfunction or that the observed deficits are induced by overexpressed monomeric αsyn. While we know that αsyn oligomers associate with mitochondria because luciferase activity is only be detected when oligomers are present, we cannot rule out the possibility that undetected monomers of αsyn are also present and responsible for some of the observed effects. We also cannot rule out the possibility that higher order αsyn species detected via native PAGE are actually representative of a protein complex containing αsyn. To address these concerns will require methods to selectively deplete cytosolic αsyn in cultured cells as well as methods to distinguish between monomers and oligomers when αsyn is expressed in cells. To our knowledge such tools have yet to be developed.

In line with our results, deficiency of SIRT3 is observed in cellular models of HD [20] and down regulation of SIRT3 increases dopaminergic cell death in an 1-methyl-4-phenyl-1,2,3,6-tetrahydropyridine (MPTP) mouse model of PD [6]. Most recently, overexpression of SIRT3 was demonstrated to prevent αsyn-induced neurodegeneration in a rodent AAV model [11]. In humans, downregulation of SIRT3 has been previously reported in post-mortem human AD brain [49, 50].

SIRT3 is emerging as an important regulator of cellular biogenesis and oxidative stress. Recent evidence supports attenuation of ROS and improved mitochondrial bioenergetics upon activation of SIRT3 [51], while SIRT3 knockdown exacerbates ROS production [52]. The current school of thought is that SIRT3 induces neuroprotection by enhancing mitochondrial biogenesis and integrity, perhaps by increasing mitochondrial DNA content and suppressing SOD activity [23, 52–54]. SIRT3 also seems to be under the control of AMPK/CREB-PGC-1α signaling pathway which is known to play a role in regulation of mitochondrial biogenesis and function, activating mitochondrial enzymes involved in antioxidant defenses and metabolism [17, 32, 55]. Here, we tested the hypothesis that the AMPK/CREB signaling pathway plays a role in αsyn-associated SIRT3 down-regulation. Decreased levels of p-AMPKα and p-CREB in the presence of αsyn were restored with AICAR, which also increased mitochondrial SIRT3 protein expression. Evidence in the literature supports an increase of SIRT3 activity directly by AICAR [56]. By contrast, previous studies confirm a role for AMPKα in the regulation of SIRT3 protein [57]. Herein, our data support the effect of AICAR being dependent on SIRT3 since knockdown of SIRT3 with siRNA precludes rescue by AICAR. Importantly, we found that activating AMPKα significantly reduced αsyn oligomers in our cellular model system. These data indicate that modulating SIRT3 levels may represent a target to alleviate αsyn-induced pathology and slow or halt αsyn-induced cellular dysfunction in Parkinson’s disease and related alpha-synucleinopathies.

Mitochondria are dynamic organelles that continuously undergo fission and fusion, processes necessary for cell survival and adaptation to changing energy requirements for cell growth, division, and distribution of mitochondria during differentiation [58]. Consistent with a previous study [59], our results demonstrate that mitochondrial dynamics are modified by the association of αsyn oligomers with mitochondria. Our data demonstrating increased recruitment of DRP1 to mitochondria and increased pDRP1 support increased mitochondria fission. Under stress conditions, DRP1 is recruited to mitochondria where it initiates mitochondrial fission and induces mitochondrial dysfunction. DRP1 activity is regulated by several post-translational modifications including phosphorylation at Serine 616 [41], which rapidly activates DRP1 and stimulates mitochondrial fission during mitosis [42, 60]. AICAR-treatment was able to restore DRP1 protein expression to control levels, improving mitochondrial function and indicating that SIRT3 plays an important role in regulating of maintenance of mitochondrial function during stress. Our results are consistent with a recent study demonstrating interaction of αsyn with ATP synthase in the mitochondria and impairment of complex I-dependent respiration [3]. Additionally, previous studies have shown that αsyn interacts with TOM20 [61], a protein required for mitochondrial protein import, and decreases its function. SIRT3 is reported to exist in the cytoplasm in an inactive form and is recruited to the mitochondria upon stress [62], although the data presented here do not support cytosolic SIRT3 expression. It is tempting to speculate that TOM20 plays a role in the translocation of SIRT3 to mitochondria and that αsyn-induced deficit in protein import results in reduced mitochondrial SIRT3 levels thereby initiating the cascade of mitochondrial dysfunction that results in decreased mitochondrial bioenergetics.

## Conclusions

Our results identify a mechanism whereby the association of αsyn oligomers with mitochondria contribute to impaired mitochondrial respiration and impaired mitochondrial dynamics by disrupting AMPK/CREB/SIRT3 signaling (Fig. 8). Taken together, our study opens the door to the use of SIRT3 activators as potential therapeutics for restoration of mitochondrial deficits and decrease in αsyn-induced pathophysiology. Further studies will be necessary to address the role of SIRT3 deacetylation substrates as possible players in PD pathogenesis.

## Supplementary information

**Additional file 1: Figure S1. (a)** Luciferase activity in cytosolic fractions of H4 SL1&SL2 cells over time, *n* = 5 **(b)** Representative cropped western blots of denaturing SDS-page (left) and native-page (right) performed at 72 h to confirm the purity of the mitochondrial-enriched fractions, the lysates were probed for the golgi marker (GM130), inner mitochondrial membrane marker (COXIV), GAPDH and αsyn in cytosol and mitochondria from H4 SL1&SL2 cells. **(c)** Representative image of dot-blot assay of the mitochondrial fractions at 72 h probed for amyloid-specific A11 and αsyn disease-associated (5G4) antibodies. **(d)** SIRT3 expression was detected by immune-fluorescence in mitochondria. SIRT3 expression is decreased at 72 h in cells overexpressing αsyn. Representative image from 3 experiments. DAPI (nucleus; blue); αsyn (green); SIRT3 (mitochondria; orange); merged images (yellow). Scale bar = 10 μm. Error bars represent the mean ± SD (*n* = 3–5). **p < 0.01.

**Additional file 2: Figure S2. (a)** Representative cropped western blots showing αsyn in cytosolic and mitochondrial fractions from H4 WT-αsyn overexpressing cells. αSyn, GAPDH, and COXIV are all from same samples and immunoblot. **(b)** Western blot and quantification for SIRT3 in primary embryonic mouse neurons treated at DIV7 for 5 days with either AAV2/8 WT-αsyn or AAV2/8 venus control. Untagged αsyn overexpression shows a significant decrease in SIRT3 level compared to control. Error bars represent the mean ± SD. **p < 0.01 (*n* = 4).

**Additional file 3: Figure S3. (a)** Representative cropped western blot showing DRP1 and p-DRP1 (Ser 616) in whole cells lysates from H4 SL1&SL2 cells over time (*n* = 4). Removal of tet leads to significantly increased p-DRP1 by 72 h. **(b)** Quantification of p-DRP1/DRP1 protein ratio in whole cell lysates. Error bars represent the mean ± SD. **p < 0.01.

**Additional file 4: Figure S4. (a)** Representative cropped western blot of lysates from dissected substantia nigra/midbrain (SN) of three rats injected unilaterally with control virus, AAV8-Hgluc (gaussia luciferase) demonstrates no change in SIRT3 protein levels when contralateral (C) uninjected SN is compared to ipsilateral (I) injected SN **(b)** Representative cropped western blot of AMPKα, p-AMPKα (Thr 172), CREB, and p-CREB (Ser 133) in SN lysate of AAV8-SL1&SL2 injected rat. αSyn expression leads to decreased p-AMPK and p-CREB protein levels in ipsilateral (I) injected SN compared to contralateral (C) uninjected SN, *n* = 5 rats total. Error bars represent the mean ± SD. *p < 0.05, **p < 0.01.

## Data Availability

All data generated or analyzed during this study are included in this published article and the supplementary information files.

## Abbreviations

AD: Alzheimer’s disease
AICAR: 5-aminoimidazole-4-carboxamide-1-β-d-ribofuranoside
AMPK: Adenosine monophosphate activated protein kinase
CREB: CAMP-response element binding protein
DRP1: Dynamin-related protein 1
HD: Huntington’s disease
HO-1: Heme oxygenase-1
LBD: Lewy body disease
mtROS: Mitochondrial reactive oxygen species
OCR: Oxygen consumption rate
PD: Parkinson disease
SIRT3: Sirtuin 3
SOD2: Superoxide dismutase 2
α-synuclein: αsyn

## References

[1] Devi L, Raghavendran V, Prabhu BM, Avadhani NG, Anandatheerthavarada HK (2008). Mitochondrial import and accumulation of alpha-synuclein impair complex I in human dopaminergic neuronal cultures and Parkinson disease brain. J Biol Chem.

[2] Hsu LJ, Sagara Y, Arroyo A, Rockenstein E, Sisk A, Mallory M, Wong J, Takenouchi T, Hashimoto M, Masliah E (2000). Alpha-synuclein promotes mitochondrial deficit and oxidative stress. Am J Pathol.

[3] Ludtmann MHR, Angelova PR, Horrocks MH, Choi ML, Rodrigues M, Baev AY, Berezhnov AV, Yao Z, Little D, Banushi B (2018). α-Synuclein oligomers interact with ATP synthase and open the permeability transition pore in Parkinson's disease. Nat Commun.

[4] Reeve AK, Ludtmann MH, Angelova PR, Simcox EM, Horrocks MH, Klenerman D, Gandhi S, Turnbull DM, Abramov AY (2015). Aggregated α-synuclein and complex I deficiency: exploration of their relationship in differentiated neurons. Cell Death Dis.

[5] Kyrylenko S, Baniahmad A (2010). Sirtuin family: a link to metabolic signaling and senescence. Curr Med Chem.

[6] Liu L, Peritore C, Ginsberg J, Kayhan M, Donmez G (2015). SIRT3 attenuates MPTP-induced nigrostriatal degeneration via enhancing mitochondrial antioxidant capacity. Neurochem Res.

[7] Liu L, Peritore C, Ginsberg J, Shih J, Arun S, Donmez G (2015). Protective role of SIRT5 against motor deficit and dopaminergic degeneration in MPTP-induced mice model of Parkinson’s disease. Behav Brain Res.

[8] Outeiro TF, Kontopoulos E, Altmann SM, Kufareva I, Strathearn KE, Amore AM, Volk CB, Maxwell MM, Rochet JC, McLean PJ (2007). Sirtuin 2 inhibitors rescue alpha-synuclein-mediated toxicity in models of Parkinson's disease. Science..

[9] Jin F, Wu Q, Lu YF, Gong QH, Shi JS (2008). Neuroprotective effect of resveratrol on 6-OHDA-induced Parkinson’s disease in rats. Eur J Pharmacol.

[10] Lofrumento DD, Nicolardi G, Cianciulli A, De Nuccio F, La Pesa V, Carofiglio V, Dragone T, Calvello R, Panaro MA (2014). Neuroprotective effects of resveratrol in an MPTP mouse model of Parkinson’s-like disease: possible role of SOCS-1 in reducing pro-inflammatory responses. Innate Immun.

[11] Gleave JA, Arathoon LR, Trinh D, Lizal KE, Giguère N, Barber JHM, Najarali Z, Khan MH, Thiele SL, Semmen MS, Koprich JB, Brotchie JM, Eubanks JH, Trudeau LE, Nash JE (2017). Sirtuin 3 rescues neurons through the stabilisation of mitochondrial biogenetics in the virally-expressing mutant α-synuclein rat model of parkinsonism. Neurobiol Dis.

[12] Hebert AS, Dittenhafer-Reed KE, Yu W, Bailey DJ, Selen ES, Boersma MD, Carson JJ, Tonelli M, Balloon AJ, Higbee AJ (2013). Calorie restriction and SIRT3 trigger global reprogramming of the mitochondrial protein acetylome. Mol Cell.

[13] Herskovits AZ, Guarente L (2013). Sirtuin deacetylases in neurodegenerative diseases of aging. Cell Res.

[14] Lombard DB, Alt FW, Cheng HL, Bunkenborg J, Streeper RS, Mostoslavsky R, Kim J, Yancopoulos G, Valenzuela D, Murphy A (2007). Mammalian Sir2 homolog SIRT3 regulates global mitochondrial lysine acetylation. Mol Cell Biol.

[15] López-Otín C, Blasco MA, Partridge L, Serrano M, Kroemer G (2013). The hallmarks of aging. Cell.

[16] Bause AS, Haigis MC (2013). SIRT3 regulation of mitochondrial oxidative stress. Exp Gerontol.

[17] Kong X, Wang R, Xue Y, Liu X, Zhang H, Chen Y, Fang F, Chang Y (2010). Sirtuin 3 a new target of PGC-1α, plays an important role in the suppression of ROS and mitochondrial biogenesis. PLoS One.

[18] Ansari A, Rahman MS, Saha SK, Saikot FK, Deep A, Kim KH (2017). Function of the SIRT3 mitochondrial deacetylase in cellular physiology, cancer, and neurodegenerative disease. Aging Cell.

[19] Kim SH, Lu HF, Alano CC (2011). Neuronal Sirt3 protects against excitotoxic injury in mouse cortical neuron culture. PLoS One.

[20] Fu J, Jin J, Cichewicz RH, Hageman SA, Ellis TK, Xiang L (2012). Trans-(−)-ε-Viniferin increases mitochondrial sirtuin 3 (SIRT3), activates AMP-activated protein kinase (AMPK), and protects cells in models of Huntington disease. J Biol Chem.

[21] Weir HJ, Murray TK, Kehoe PG, Love S, Verdin EM, O'Neill MJ (2012). CNS SIRT3 expression is altered by reactive oxygen species and in Alzheimer's disease. PLoS One.

[22] Yin J, Han P, Tang Z, Liu Q, Shi J (2015). Sirtuin 3 mediates neuroprotection of ketones against ischemic stroke. J Cereb Blood Flow Metab.

[23] Liu J, Li D, Zhang T, Tong Q, Ye RD, Lin L (2017). SIRT3 protects hepatocytes from oxidative injury by enhancing ROS scavenging and mitochondrial integrity. Cell Death Dis.

[24] Moussaud S, Malany S, Mehta A, Vasile S, Smith LH, McLean PJ (2015). Targeting α-synuclein oligomers by protein-fragment complementation for drug discovery in synucleinopathies. Expert Opin Ther Targets.

[25] Delenclos M, Trendafilova T, Jones DR, Moussaud S, Baine AM, Yue M, Hirst WD, McLean PJ (2016). A Rapid, Semi-Quantitative Assay to Screen for Modulators of Alpha-Synuclein Oligomerization Ex vivo. Front Neurosci.

[26] Paxinos G, Watson C (1998). The rat brain in stereotaxic coordinates.

[27] Marongiu R, Spencer B, Crews L, Adame A, Patrick C, Trejo M (2009). Mutant Pink1 induces mitochondrial dysfunction in a neuronal cell model of Parkinson’s disease by disturbing calcium flux. J Neurochem.

[28] Nakamura K, Nemani VM, Azarbal F, Skibinski G, Levy JM, Egami K, Munishkina L, Zhang J, Gardner B, Wakabayashi J, Sesaki H, Cheng Y, Finkbeiner S, Nussbaum RL, Masliah E, Edwards RH (2011). Direct membrane association drives mitochondrial fission by the Parkinson disease-associated protein alpha-synuclein. J Biol Chem.

[29] Kayed R, Head E, Thompson JL, McIntire TM, Milton SC, Cotman CW, Glabe CG (2003). Common structure of soluble amyloid oligomers implies common mechanism of pathogenesis. Science.

[30] Kovacs GG, Wagner U, Dumont B, Pikkarainen M, Osman AA, Streichenberger N, Leisser I, Verchère J, Baron T, Alafuzoff I, Budka H, Perret-Liaudet A, Lachmann I (2012). An antibody with high reactivity for disease-associated α-synuclein reveals extensive brain pathology. Acta Neuropathol.

[31] Pillai VB, Sundaresan NR, Kim G, Gupta M, Rajamohan SB, Pillai JB (2010). Exogenous NAD blocks cardiac hypertrophic response via activation of the SIRT3-LKB1-AMP-activated kinase pathway. J Biol Chem.

[32] Shi T, Wang F, Stieren E, Tong Q (2005). SIRT3, a mitochondrial sirtuin deacetylase, regulates mitochondrial function and thermogenesis in brown adipocytes. J Biol Chem.

[33] Dulovic M, Jovanovic M, Xilouri M, Stefanis L, Harhaji-Trajkovic L, Kravic-Stevovic T, Paunovic V, Ardah MT, El-Agnaf OM, Kostic V, Markovic I, Trajkovic V (2014). The protective role of AMP-activated protein kinase in alpha-synuclein neurotoxicity in vitro. Neurobiol Dis.

[34] Takeuchi K, Morizane Y, Kamami-Levy C, Suzuki J, Kayama M, Cai W (2013). AMP-dependent kinase inhibits oxidative stress-induced caveolin-1 phosphorylation and endocytosis by suppressing the dissociation between c-Abl and Prdx1 proteins in endothelial cells. J Biol Chem.

[35] Qiu X, Brown K, Hirschey MD, Verdin E, Chen D (2010). Calorie restriction reduces oxidative stress by SIRT3-mediated SOD2 activation. Cell Metab.

[36] Torrens-Mas M, Oliver J, Roca P, Sastre-Serra J (2017). SIRT3: Oncogene and Tumor Suppressor in Cancer. Cancers (Basel).

[37] Bansal S, Biswas G, Avadhani NG (2013). Mitochondria-targeted heme oxygenase-1 induces oxidative stress and mitochondrial dysfunction in macrophages, kidney fibroblasts and in chronic alcohol heaptotoxicity. Redox Biol.

[38] Shipper HM, Liberman A, Stopa EG (1998). Neural heme oxygenase-1 expression in idiopathic Parkinson's disease. Exp Neurol.

[39] Song W, Patel A, Qureshi HY, Han D, Schipper HM, Paudel HK (2009). The Parkinson disease-associated A30P mutation stabilizes alpha-synuclein against proteasomal degradation triggered by heme oxygenase-1 over-expression in human neuroblastoma cells. J Neurochem.

[40] Alaimo A, Gorojod RM, Beauquis J, Muñoz MJ, Saravia F, Kotler ML (2014). Deregulation of mitochondria-shaping proteins Opa-1 and Drp-1 in manganese-induced apoptosis. PLoS One.

[41] Elgass K, Pakay J, Ryan MT, Palmer CS (1833). Recent advances into the understanding of mitochondrial fission. Biochim Biophys Acta.

[42] Taguchi N, Ishihara N, Jofuku A, Oka T, Mihara K (2007). Mitotic phosphorylation of dynamin-related GTPase Drp1 participates in mitochondrial fission. J Biol Chem.

[43] Dawson TM, Dawson VL (2003). Molecular pathways of neurodegeneration in Parkinson’s disease. Science..

[44] Schapira AH, Hartley A, Cleeter MW, Cooper JM (1993). Free radicals and mitochondrial dysfunction in Parkinson's disease. Biochem Soc Trans.

[45] Bobela W, Nazeeruddin S, Knott G, Aebischer P, Schneider BL (2017). Modulating the catalytic activity of AMPK has neuroprotective effects against α-synuclein toxicity. Mol Neurodegener.

[46] Siddiqui A, Chinta SJ, Mallajosyula JK, Rajagopolan S, Hanson I, Rane A, Melov S, Andersen JK (2012). Selective binding of nuclear alpha-synuclein to the PGC1alpha promoter under conditions of oxidative stress may contribute to losses in mitochondrial function: implications for Parkinson's disease. Free Radic Biol Med.

[47] Sarafian TA, Ryan CM, Souda P, Masliah E, Kar UK, Vinters HV, Mathern GW, Faull KF, Whitelegge JP, Watson JB (2013). Impairment of mitochondria in adult mouse brain overexpressing predominantly full-length, N-terminally acetylated human α-synuclein. PLoS One.

[48] Subramaniam SR, Vergnes L, Franich NR, Reue K, Chesselet MF (2014). Region specific mitochondrial impairment in mice with widespread overexpression of alpha-synuclein. Neurobiol Dis.

[49] Han P, Tang Z, Yin J, Maalouf M, Beach TG, Reiman EM, Shi J (2014). Pituitary adenylate cyclase-activating polypeptide protects against β-amyloid toxicity. Neurobiol Aging.

[50] Lee J, Kim Y, Liu T, Hwang YJ, Hyeon SJ, Im H, Lee K, Alvarez VE, McKee AC, Um SJ (2018). SIRT3 deregulation is linked to mitochondrial dysfunction in Alzheimer's disease. Aging Cell.

[51] Ramesh S, Govindarajulu M, Lynd T, Briggs G, Adamek D, Jones E, Heiner J, Majrashi M, Moore T, Amin R, Suppiramaniam V, Dhanasekaran M (2018). SIRT3 activator Honokiol attenuates β-amyloid by modulating amyloidogenic pathway. PLoS One.

[52] Zhang JY, Deng YN, Zhang M, Su H, Qu QM (2016). SIRT3 acts as a Neuroprotective agent in rotenone-induced Parkinson cell model. Neurochem Res.

[53] Dai SH, Chen T, Wang YH, Zhu J, Luo P, Rao W, Yang YF, Fei Z, Jiang XF (2014). Sirt3 protects cortical neurons against oxidative stress via regulating mitochondrial Ca2^+^ and mitochondrial biogenesis. Int J Mol Sci.

[54] Dai SH, Chen T, Wang YH, Zhu J, Luo P, Rao W, Yang YF, Fei Z, Jiang XF (2014). Sirt3 attenuates hydrogen peroxide induced oxidative stress through the preservation of mitochondrial function in HT22 cells. Int J Mol Med.

[55] Abdel Khalek Waed, Cortade Fabienne, Ollendorff Vincent, Lapasset Laure, Tintignac Lionel, Chabi Béatrice, Wrutniak-Cabello Chantal (2014). SIRT3, a Mitochondrial NAD+-Dependent Deacetylase, Is Involved in the Regulation of Myoblast Differentiation. PLoS ONE.

[56] Morigi Marina, Perico Luca, Rota Cinzia, Longaretti Lorena, Conti Sara, Rottoli Daniela, Novelli Rubina, Remuzzi Giuseppe, Benigni Ariela (2015). Sirtuin 3–dependent mitochondrial dynamic improvements protect against acute kidney injury. Journal of Clinical Investigation.

[57] Brandauer J, Andersen MA, Kellezi H, Risis S, Frøsig C, Vienberg SG, Treebak JT. AMP-activated protein kinase controls exercise training- and AICAR-induced increases in SIRT3 and MnSOD. Front Physiol. 2015;6:85. 10.3389/fphys.2015.00085 PMC4371692 25852572

[58] van der Bliek AM, Shen Q, Kawajiri S. Mechanisms of mitochondrial fission and fusion. Cold Spring Harb Perspect Biol. 2013;5:a011072. 10.1101/cshperspect.a011072 PMC3660830 23732471

[59] Martinez Jimena Hebe, Alaimo Agustina, Gorojod Roxana Mayra, Porte Alcon Soledad, Fuentes Federico, Coluccio Leskow Federico, Kotler Mónica Lidia (2018). Drp-1 dependent mitochondrial fragmentation and protective autophagy in dopaminergic SH-SY5Y cells overexpressing alpha-synuclein. Molecular and Cellular Neuroscience.

[60] Sanchis-Gomar F, Derbré F. Mitochondrial fission and fusion in human diseases. N Engl J Med. 2014;370:1073-4. 10.1056/NEJMc1316254 24620885

[61] Di Maio R, Barrett PJ, Hoffman EK, Barrett CW, Zharikov A, Borah A, Hu X, McCoy J, Chu CT, Burton EA, Hastings TG, Greenamyre JT. α- Synuclein binds to TOM20 and inhibits mitochondrial protein import in Parkinson's disease. Sci Transl. 2016;8:342ra78. 10.1126/scitranslmed.aaf3634 PMC5016095 27280685

[62] Anamika, Khanna Archita, Acharjee Papia, Acharjee Arup, Trigun Surendra Kumar (2019). Mitochondrial SIRT3 and neurodegenerative brain disorders. Journal of Chemical Neuroanatomy.

