# Supplementary material for: Alpha-synuclein-induced mitochondrial dysfunction is mediated via a sirtuin 3-dependent pathway
Source: Mol Neurodegener. 2020 Jan 13;15:5. doi: 10.1186/s13024-019-0349-x (PMC6956494; doi:10.1186/s13024-019-0349-x)
Supplement: Supplementary file 1 — Additional file 1: Figure S1. (a) Luciferase activity in cytosolic fractions of H4 SL1&SL2 cells over time, n = 5 (b) Representative cropped western blots of denaturing SDS-page (left) and native-page (right) performed at 72 h to confirm the purity of the mitochondrial-enriched fractions, the lysates were probed for the golgi marker (GM130), inner mitochondrial membrane marker (COXIV), GAPDH and αsyn in cytosol and mitochondria from H4 SL1&SL2 cells. (c) Representative image of dot-blot assay of the mitochondrial fractions at 72 h probed for amyloid-specific A11 and αsyn disease-associated (5G4) antibodies. (d) SIRT3 expression was detected by immune-fluorescence in mitochondria. SIRT3 expression is decreased at 72 h in cells overexpressing αsyn. Representative image from 3 experiments. DAPI (nucleus; blue); αsyn (green); SIRT3 (mitochondria; orange); merged images (yellow). Scale bar = 10 μm. Error bars represent the mean ± SD (n = 3–5). **p < 0.01. [file 13024_2019_349_MOESM1_ESM.docx]

**Figure S1**

**
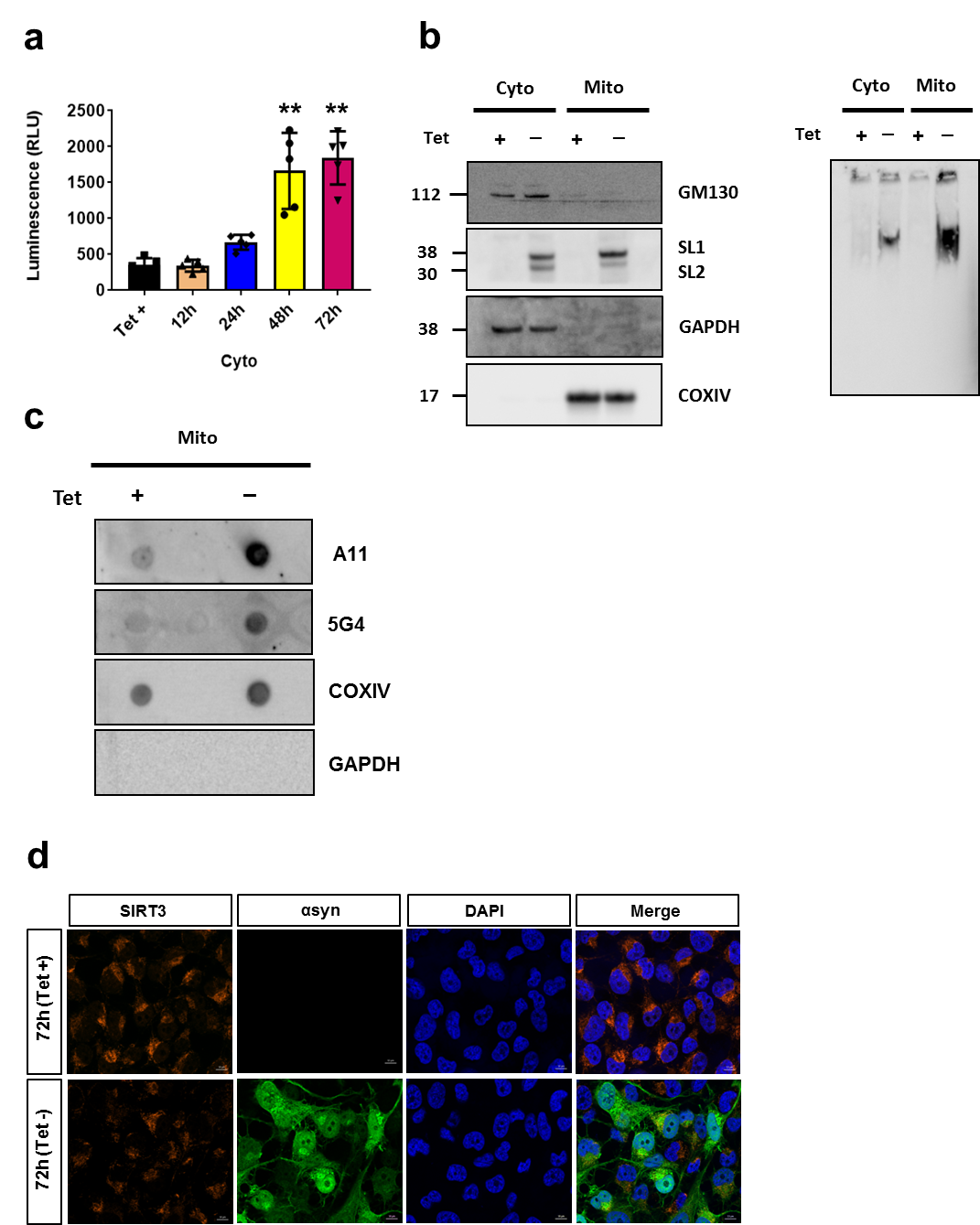
**

**Figure S1: (a)** Luciferase activity in cytosolic fractions of H4 SL1&SL2 cells over time, n=5 **(b)** Representative cropped western blots western blots of denaturing SDS-page (n=4) and native-page performed at 72h to confirm the purity of the mitochondrial-enriched fractions, the lysates were probed for the golgi marker (GM130), inner mitochondrial membrane marker (COXIV), GAPDH, total αsyn levels, and αsyn oligomers in cytosol and mitochondria from H4 SL1&SL2 cells. **(c)** Representative image of α-syn oligomer dot-blot assay at 72h with amyloid-specific antibody A11 and αsyn disease-associated 5G4 antibody. **(d)** SIRT3 expression was detected by immunofluorescence in mitochondria. SIRT3 expression is decreased at 72h in cells overexpressing αsyn. Representative image from 3 experiments. DAPI (nucleus; blue); αsyn (green); SIRT3 (mitochondria; orange); merged images (yellow). Scale bar = 10 μm. Error bars represent the mean ± SD (n = 3-5). **p < 0.01.
