# Supplementary material for: Alpha-synuclein-induced mitochondrial dysfunction is mediated via a sirtuin 3-dependent pathway
Source: Mol Neurodegener. 2020 Jan 13;15:5. doi: 10.1186/s13024-019-0349-x (PMC6956494; doi:10.1186/s13024-019-0349-x)
Supplement: Supplementary file 2 — Additional file 2: Figure S2. (a) Representative cropped western blots showing αsyn in cytosolic and mitochondrial fractions from H4 WT-αsyn overexpressing cells. αSyn, GAPDH, and COXIV are all from same samples and immunoblot. (b) Western blot and quantification for SIRT3 in primary embryonic mouse neurons treated at DIV7 for 5 days with either AAV2/8 WT-αsyn or AAV2/8 venus control. Untagged αsyn overexpression shows a significant decrease in SIRT3 level compared to control. Error bars represent the mean ± SD. **p < 0.01 (n = 4). [file 13024_2019_349_MOESM2_ESM.docx]

**Figure S2**

**
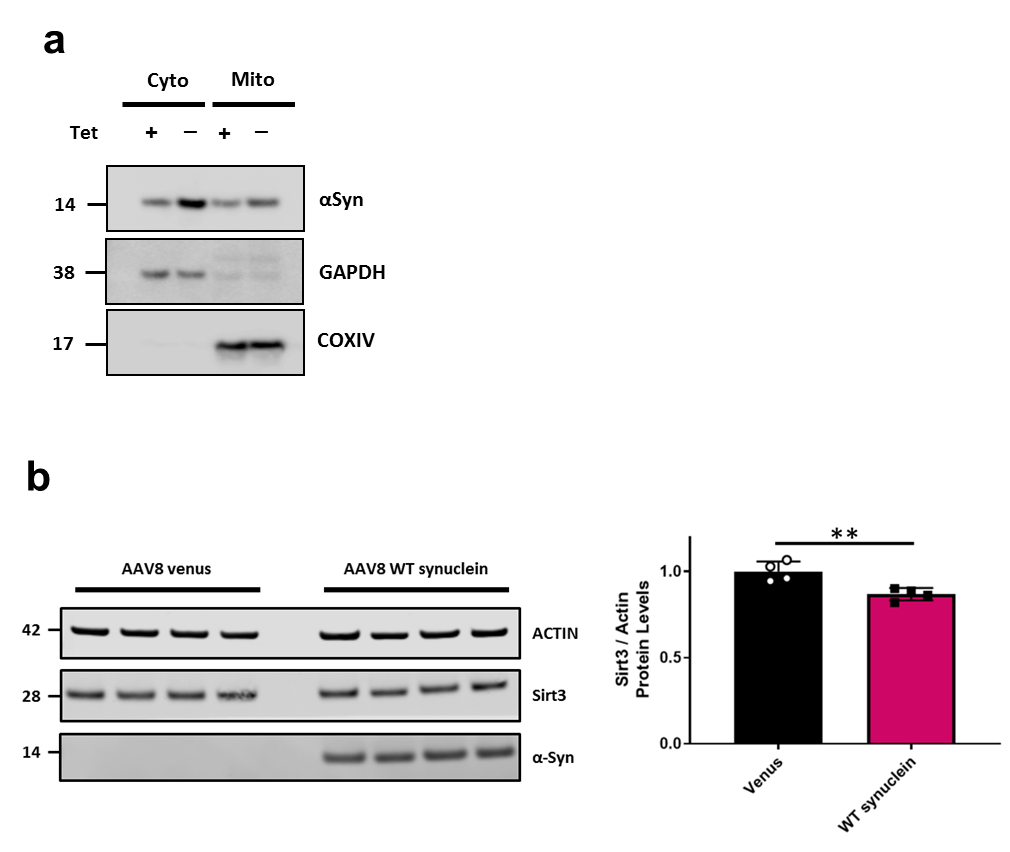
**

**Figure S2: (a)** Representative cropped western blots showing αsyn in cytosolic and mitochondrial fractions from H4 WT-αsyn overexpressing cells. αSyn, GAPDH, and COXIV are all from same samples and immunoblot. **(b)** Western blot and quantification for SIRT3 in primary embryonic mouse neurons treated at DIV7 for 5 days with either AAV2/8 WT-αsyn or AAV2/8 venus control. Untagged αsyn overexpression shows a significant decrease in SIRT3 level compared to control. Error bars represent the mean ± SD. **p < 0.01 (n=4).
