# Supplementary material for: Alpha-synuclein-induced mitochondrial dysfunction is mediated via a sirtuin 3-dependent pathway
Source: Mol Neurodegener. 2020 Jan 13;15:5. doi: 10.1186/s13024-019-0349-x (PMC6956494; doi:10.1186/s13024-019-0349-x)
Supplement: Supplementary file 3 — Additional file 3: Figure S3. (a) Representative cropped western blot showing DRP1 and p-DRP1 (Ser 616) in whole cells lysates from H4 SL1&SL2 cells over time (n = 4). Removal of tet leads to significantly increased p-DRP1 by 72 h. (b) Quantification of p-DRP1/DRP1 protein ratio in whole cell lysates. Error bars represent the mean ± SD. **p < 0.01. [file 13024_2019_349_MOESM3_ESM.docx]

**Figure S3**

**
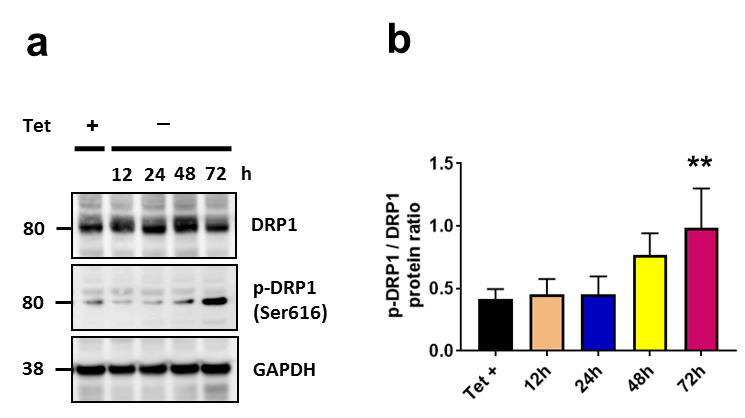
**

**Figure S3: (a)** Representative cropped western blot from showing DRP1 and p-DRP1 (Ser 616) in whole cells lysates from H4 SL1&SL2 cells over time (n=4). Removal of tet leads to significantly increased p-DRP1 by 72h. **(b)** Quantification of p-DRP1/DRP1 protein ratio in whole cell lysates. Error bars represent the mean ± SD. **p < 0.01.
