# Supplementary material for: Alpha-synuclein-induced mitochondrial dysfunction is mediated via a sirtuin 3-dependent pathway
Source: Mol Neurodegener. 2020 Jan 13;15:5. doi: 10.1186/s13024-019-0349-x (PMC6956494; doi:10.1186/s13024-019-0349-x)
Supplement: Supplementary file 4 — Additional file 4: Figure S4. (a) Representative cropped western blot of lysates from dissected substantia nigra/midbrain (SN) of three rats injected unilaterally with control virus, AAV8-Hgluc (gaussia luciferase) demonstrates no change in SIRT3 protein levels when contralateral (C) uninjected SN is compared to ipsilateral (I) injected SN (b) Representative cropped western blot of AMPKα, p-AMPKα (Thr 172), CREB, and p-CREB (Ser 133) in SN lysate of AAV8-SL1&SL2 injected rat. αSyn expression leads to decreased p-AMPK and p-CREB protein levels in ipsilateral (I) injected SN compared to contralateral (C) uninjected SN, n = 5 rats total. Error bars represent the mean ± SD. *p < 0.05, **p < 0.01. [file 13024_2019_349_MOESM4_ESM.docx]

**Figure S4**

**
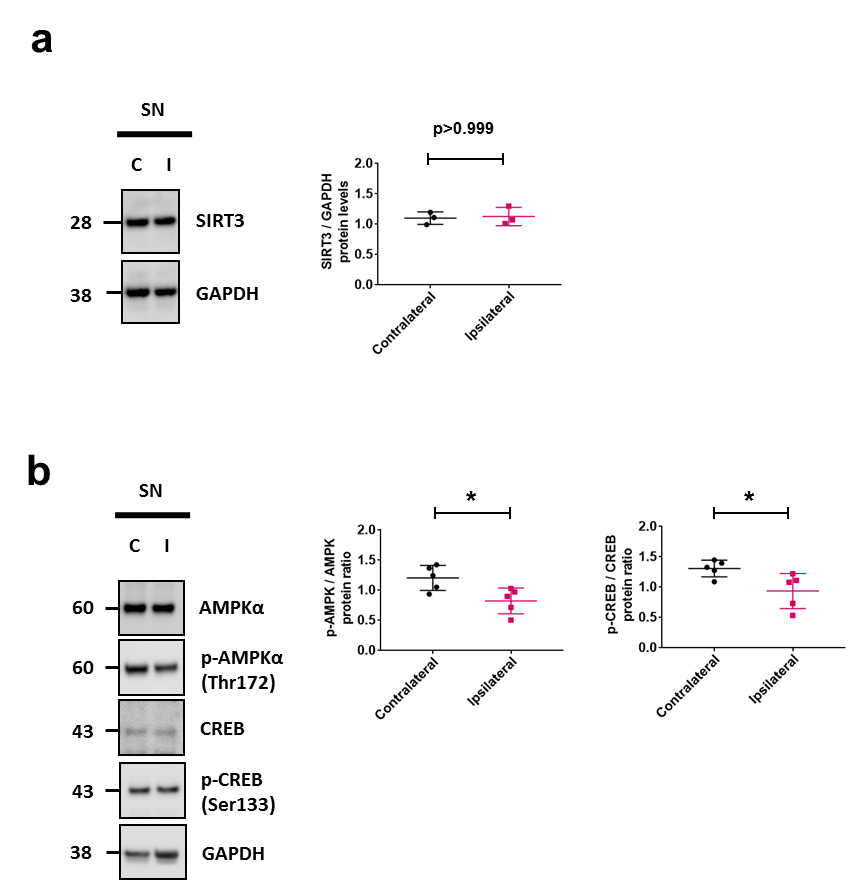
**

**Figure S4:**  **(a)** Representative cropped western blot of lysates from dissected substantia nigra/midbrain (SN) of three rats injected unilaterally with control virus, AAV8-Hgluc (*gaussia luciferase*) demonstrates no change in SIRT3 protein levels when contralateral (C) uninjected SN is compared to ipsilateral (I) injected SN **(b)** Representative cropped western blot of AMPKα, p-AMPKα (Thr 172), CREB, and p-CREB (Ser 133) in SN lysate of AAV8-SL1&SL2 injected rat. αSyn expression leads to decreased p-AMPK and p-CREB protein levels in ipsilateral (I) injected SN compared to contralateral (C) uninjected SN, n=5 rats total. Error bars represent the mean ± SD. *p < 0.05, **p < 0.01.
